# Supplementary material for: Active site specificity profiling datasets of matrix metalloproteinases (MMPs) 1, 2, 3, 7, 8, 9, 12, 13 and 14
Source: Data Brief. 2016 Feb 22;7:299–310. doi: 10.1016/j.dib.2016.02.036 (PMC4777984; doi:10.1016/j.dib.2016.02.036)
Supplement: Supplementary file 10 — Supplementary material [file mmc10.zip › WebPICS_hMMP1_G_1%/P2prime.html]

 

PICS results


|  |  |
| --- | --- |
| **P2prime\_A**  12 in 126 sites   9.5 %    effects > 10 perc. pnts.  (vice-versa in brackets)  P2\_C: 13.5 (40.5) |  |
| **P2prime\_F**  6 in 126 sites   4.8 %    effects > 10 perc. pnts.  (vice-versa in brackets)  P2\_Y: 40.5 (20.2)   P1\_A: 24.6 (13.4)   P3prime\_V: 37.3 (14.0) |  |
| **P2prime\_I**  12 in 126 sites   9.5 %    effects > 10 perc. pnts.  (vice-versa in brackets)  P2\_K: 10.4 (15.5)   P3prime\_N: 24.6 (26.9) |  |
| **P2prime\_K**  23 in 126 sites   18.3 %    effects > 10 perc. pnts.  (vice-versa in brackets)  P3\_V: 17.7 (25.5)   P1\_H: 11.8 (38.8) |  |
| **P2prime\_L**  16 in 126 sites   12.7 %    effects > 10 perc. pnts.  (vice-versa in brackets)  P3\_A: 18.5 (12.3)   P2\_L: 12.3 (12.3)   P2\_Q: 20.1 (23.0)   P1\_A: 16.3 (23.7)   P1\_Y: 13.2 (30.2)   P3prime\_K: 10.9 (17.3)   P3prime\_Q: 16.3 (23.7) |  |
| **P2prime\_Q**  5 in 126 sites   4.0 %    effects > 10 perc. pnts.  (vice-versa in brackets)  P1\_H: 14.4 (10.3) |  |
| **P2prime\_T**  10 in 126 sites   7.9 %    effects > 10 perc. pnts.  (vice-versa in brackets)  P3\_T: 11.3 (10.3)   P2\_A: 30.5 (25.4)   P2\_N: 25.2 (42.1)   P1prime\_C: 23.7 (29.6)   P3prime\_T: 21.3 (19.4)   P3prime\_V: 17.3 (10.9) |  |
| **P2prime\_V**  13 in 126 sites   10.3 %    effects > 10 perc. pnts.  (vice-versa in brackets)  P3\_P: 26.4 (13.7)   P3\_T: 14.4 (17.0)   P2\_Y: 13.6 (14.7)   P1\_P: 10.6 (23.0)   P1\_S: 15.7 (10.8) |  |
| **P2prime\_Y**  4 in 126 sites   3.2 %    effects > 10 perc. pnts.  (vice-versa in brackets)  P2\_M: 20.2 (13.5)   P2\_Y: 40.5 (13.5)   P1\_P: 20.2 (13.5)   P3prime\_V: 62.3 (15.6) |  |
